# Supplementary material for: Policy and behavioral response to shock events: An agent-based model of the effectiveness and equity of policy design features
Source: PLoS One. 2022 Jan 21;17(1):e0262172. doi: 10.1371/journal.pone.0262172 (PMC8782474; doi:10.1371/journal.pone.0262172)
Supplement: S1 Appendix — (DOCX) [file pone.0262172.s001.docx]

**S1 Appendix: Model parameters and equations**

**Scheduling**

Key aspects of agent progression and state duration are drawn from recent literature in epidemiology (Tec et al. 2020). This ABM uses age-dependent progression probabilities as follows:

Pr(Iy to Rr | age < 49) = 0.967

Pr(Iy to Rd | age < 49) = 1-Pr(Iy to Rr | age < 49)

Pr(Iy to Rr | age >= 49) = 0.887

Pr(Iy to Rd | age >= 49) = 1-Pr(Iy to Rr | age >= 49)

Pr(Iy to Rr | age >=64) = 0.823

Pr(Iy to Rd | age >=64) = 1-Pr(Iy to Rr | age >=64)

Pr(Ih to Rr | age < 49) = 0.969

Pr(Ih to Rd | age < 49) = 1-Pr(Ih to Rr | age < 49)

Pr(Ih to Rr | age >= 49) = 0.893

Pr(Ih to Rd | age >= 49) = 1-Pr(Ih to Rr | age >= 49)

Pr(Ih to Rr | age >=64) = 0.769

Pr(Ih to Rd | age >=64) = 1-Pr(Ih to Rr | age >=64)

The remaining progression probabilities, applied to all agents regardless of age, are:

Pr(E to Pa) = 0.83

Pr(E to Py) = 1-Pr(E to Pa)

Pr(Ia to Rr) = 1

Pr(Iy to Rr) = 0.935

Pr(Iy to Ih) = 1-Pr(Iy to Rr)

Agents spend a random duration in each compartment they enter. Transitions between compartments are modelled as the inter-arrival time of a Poisson arrival process per Equation A1.

$duration=-ln(1-\gamma)/\lambda$, A1

where $\gamma$ is a random variable distributed ~ U(0,1), and $\lambda$ is the relevant state duration estimated in the epidemiological literature. Those values of $\lambda$, measured in days, are:

Duration E: 2.9

Duration Pa: 2.3

Duration Py: 2.3

Duration Ia: 4

Duration Iy transitioning to Ih: 5.9

Duration Iy transitioning to Rr: 4

Duration Ih transitioning to Rr: 10.9

Duration Ih transitioning to Rd: 7.8

Agents also schedule potential exposure events in each of the spheres, conditional on their infection state. These time between these events are also modelled as the inter-arrival time of a Poisson arrival process per Equation A1. Those values of $\lambda$, measured in days, are:

Essential activity, receiving: 3/7

Non-essential (social) activity: 10/7

Essential activity, providing: 5/7

**Interaction and Exposure**

When agents interact, they have asymmetric probability of infecting and becoming infected. An infected agent involved in an interaction has a probability of exposing susceptible agents involved in the same interaction per the following equations.

$baseline=\frac{transmissibility}{1-transmissibility}$ , A2

$own=\frac{riskTolerance}{1-riskTolerance}$ , A3

$local=\frac{mean(localRisk)}{1-mean(localRisk)}$, and A4

$\Pr\left( Event \right)=\frac{baseline+own+local}{1+(baseline+own+local)}$ . A5

where we assume a transmissibility parameter of 0.5, *riskTolerance* is the risk tolerance of the **infected** agent, and *localRisk* is the set of *riskTolerance* for all other agents involved in the same interaction. When a Bernoulli draw with pr(1)=pr(Event), a potential exposure occurs. However, the probability of an agent becoming exposed – and moving into the E compartment is asymmetric per the same set of equations, but with *riskTolerance* as the risk tolerance of any **susceptible** agent. When a potential exposure occurs *and* a Bernoulli draw with pr(1)=pr(Event) relative to the susceptible agent, that agent becomes exposed.

**Risk Tolerance Updates**

Agents update their risk tolerance at the beginning of each time simulated day. Updates are performed per:

${RiskTolerance}_{i}= \frac{exp(\beta_{0}+\beta_{1}{Age}_{i}+\beta_{2}{SickFrac}_{i})}{1+exp(\beta_{0}+\beta_{1}{Age}_{i}+\beta_{2}{SickFrac}_{i})}$ , A6

where ${RiskTolerance}_{i}$ is the updated risk tolerance for agent *i*, *Age_i_* is agent *i*’s age, *SicFrac_i_* is the proportion of agents in agent *i*’s social network that are symptomatic or hospitalized, and the beta coefficients are free parameters with the values 0.3, -0.02, and -1 respectively.
