# Supplementary material for: Policy and behavioral response to shock events: An agent-based model of the effectiveness and equity of policy design features
Source: PLoS One. 2022 Jan 21;17(1):e0262172. doi: 10.1371/journal.pone.0262172 (PMC8782474; doi:10.1371/journal.pone.0262172)
Supplement: Attachment — Submitted filename: PONE_30912_review.docx [file pone.0262172.s002.docx]

The paper “Policy and behavioral response to shock events: An agent-based model of the effectiveness and equity of policy design features” develops an agent-based model that allows for the evaluation of shelter-in-place (SIP) policies designed against the COVID-19 pandemic. The authors show, for instance, that the impact of timing of the SIP policy is moderated by other aspects of policy design and societal aspects. In my opinion, the paper makes a very important contribution on a relevant subject employing solid methods. For this reason, it deserves publication. However, the authors should perform some minor changes in the paper, as I list below:

- The Introduction of the paper is too short. The authors should extend it, elaborating on the purpose of the paper and the methods employed, as well as anticipating some results. The “COVID-19 Policy Evaluation” (CoPE), mentioned for the first time on page 11, should be briefly described in the introduction and mentioned in the abstract.
- On page 8, the expression “an SIP” should be replaced by “a SIP”.
- A more formal description of the model (CoPE), including equations and parameters, should be provided by the authors. It could be done in a separate section or an appendix.
- I suggest to the authors provide a summary table displaying all the different configurations of the SIP policy to be tested. For instance, duration: baseline (45 days), short (30 days), and long (60 days). This would improve the comprehensibility of the paper.
- On page 13, I suppose “Figure 2” should be replaced by “Figure 1”.
- On page 13, in the caption of Figure 1, both PA and PY stand for “pre symptomatic”.
- On page 14, the expression “within in the same profession” should be replaced by “within the same profession”.
- Throughout the paper, the authors use both “shelter-in-place” and “shelter in place”. Please choose just one form.
- On page 14, I suppose “Figure 2” should be replaced by “Figure 3”.
- On page 15, in the caption of Figure 3, “Compliant” should be replaced by “compliant”.
